# Microplastics in human ovarian follicular fluid: a pilot Raman spectroscopy study

**DOI:** 10.3389/fendo.2026.1856825

**Published:** 2026-09-08

**Authors:** Kálmán Kovács, Réka A. Vass, Leila Takács, József Bódis, András Lukács, Szilvia Barkó

**Affiliations:** 1 Department of Obstetrics and Gynecology, Medical School, University of Pécs, Pécs, Hungary; 2 National Laboratory on Human Reproduction, University of Pécs, Pécs, Hungary; 3 Department of Biophysics, Medical School, University of Pécs, Pécs, Hungary

**Keywords:** endocrine disruption, follicular fluid, IVF, microplastics, ovarian microenvironment, Raman spectroscopy

## Abstract

**Background:**

Microplastics (MPs) have recently been identified in multiple human biological matrices, including placenta, blood, breast milk, semen, and testicular tissue, raising concerns regarding their potential endocrine, inflammatory, and reproductive effects. Despite this rapidly expanding field, the ovarian follicular microenvironment remains largely understudied, and data on the direct presence of MPs in human follicular fluid (FF) are limited.

**Objective:**

This pilot study aimed to qualitatively assess the presence and polymer composition of MPs in human FF obtained during *in vitro* fertilization (IVF) using a contamination-controlled workflow and confocal micro-Raman spectroscopy.

**Methods:**

FF samples from 27 women undergoing IVF were processed under strict contamination-control conditions. Organic material was digested using 10% potassium hydroxide (KOH), followed by vacuum filtration through 1.6 µm glass-fiber filters. Filters were screened by optical microscopy, and suspected particles were analyzed using 532 nm confocal micro-Raman spectroscopy and matched against validated polymer spectral libraries. Procedural blanks were included in each analytical batch.

**Results:**

MPs were detected in 22 of 27 FF samples (81.5%). Positive samples contained between one and three particles. Identified polymers included polypropylene (PP; most frequent), polyethylene (PE), polyamide (PA), polystyrene (PS), and polyethylene terephthalate (PET). No MPs were detected in procedural blanks.

**Conclusions:**

MPs were detectable in the majority of FF samples under contamination-controlled conditions. While the clinical implications remain unclear, these findings support the presence of MPs within the ovarian microenvironment and highlight the need for larger, standardized, and mechanistic studies.

## 1 Introduction

Plastics have become ubiquitous in modern society due to their durability, versatility, and low production cost. However, their widespread use and inadequate waste management have led to the accumulation of microplastics (MPs), typically defined as plastic particles smaller than 5 mm, in both environmental and biological systems (1). Human exposure to MPs occurs primarily through ingestion and inhalation, with emerging evidence demonstrating their presence in multiple biological matrices, including placenta, blood, breast milk, semen, and testicular tissue (1–4). These findings have raised concerns regarding the potential endocrine, inflammatory, and reproductive effects of MPs in humans.

The reproductive system is considered particularly vulnerable to environmental stressors because of its dependence on tightly regulated endocrine and cellular interactions. In females, the ovarian follicle represents a highly specialized microenvironment in which hormonal signaling, granulosa and theca cell function, redox balance, and metabolic processes converge to support oocyte maturation (5). Disruption of this finely tuned system—even at subtle levels—may impair oocyte competence, fertilization, and subsequent embryo development. Experimental studies suggest that MPs may interfere with these processes through multiple mechanisms, including oxidative stress, mitochondrial dysfunction, endocrine disruption, and inflammatory activation (6–8).

Preclinical data provide important mechanistic insights into the potential reproductive toxicity of MPs. In rodent models, exposure to polystyrene (PS) MPs has been associated with impaired oocyte maturation, increased reactive oxygen species (ROS) production, endoplasmic reticulum stress, and granulosa cell apoptosis (6, 7). Similar findings have been reported in bovine oocyte models, where MPs disrupt spindle organization, chromosome alignment, and mitochondrial function, ultimately reducing developmental competence (8). Although these studies often involve exposure levels higher than those expected in humans, they identify biological pathways that may be relevant to human folliculogenesis.

Recent studies have demonstrated that MPs are detectable in human ovarian FF and have begun to investigate their potential clinical significance (9–11). Montano et al. first reported MPs in FF from women undergoing IVF, while subsequent studies using complementary analytical techniques confirmed these findings and suggested possible associations with ovarian reserve and reproductive function (9–11). However, despite these important advances, the available evidence remains limited by relatively small study populations and considerable methodological heterogeneity. Differences in sample collection, contamination-control procedures, analytical platforms, particle-size detection limits, polymer identification criteria, and reporting standards currently limit direct comparison among studies and hinder the reproducibility of published findings (12).

Methodological standardization therefore remains one of the major challenges in human microplastic (MP) research. Reliable detection and characterization of MPs in biological samples require rigorous contamination-control procedures because environmental and laboratory-derived particles can easily confound analytical results (12). Furthermore, analytical techniques including micro-Raman spectroscopy, micro-Fourier transform infrared spectroscopy (µFTIR), laser direct infrared spectroscopy (LD-IR), scanning electron microscopy coupled with energy-dispersive X-ray spectroscopy (SEM/EDX), and pyrolysis–gas chromatography/mass spectrometry (Py-GC/MS) each possess distinct advantages and limitations regarding sensitivity, chemical specificity, particle-size detection, and susceptibility to contamination (12). Consequently, transparent quality assurance and quality control (QA/QC) procedures, including contamination-controlled sampling, minimized plastic exposure, procedural blanks, and standardized reporting criteria, are essential to generate reliable and comparable data across studies (12, 15).

Within this context, the primary objective of the present study was not to establish the presence of MPs in human FF, which has already been demonstrated by several independent investigations (9–11), but to evaluate the feasibility of a rigorously contamination-controlled confocal micro-Raman spectroscopy workflow for qualitative polymer identification in FF obtained during IVF. By applying strict QA/QC procedures and providing transparent methodological reporting, this pilot study aims to contribute to the ongoing harmonization of analytical approaches in reproductive MP research and to provide a methodological foundation for future multicenter studies integrating quantitative exposure assessment, mechanistic biomarkers, and clinical reproductive outcomes.

## 2 Materials and methods

### 2.1 Study design, setting, and ethical considerations

This exploratory pilot study included 27 women undergoing IVF at a tertiary university-affiliated reproductive center. FF was collected during routine transvaginal oocyte retrieval. Only residual clinical material was used for analysis. The study was approved by the Regional and Local Research Ethics Committee of the University of Pécs, Hungary (approval number PTE KK 10087-PTE2025). Written informed consent was obtained from all participants. All participants provided written informed consent, and the study was conducted in accordance with institutional ethical requirements. The study was designed as a presence-detection analysis focused on qualitative polymer identification rather than quantitative burden assessment or clinicopathological association analysis.

Inclusion and exclusion criteria

Inclusion criteria comprised women undergoing controlled ovarian stimulation for IVF who provided residual FF for analysis following routine clinical processing. Exclusion criteria included conditions expected to substantially alter FF composition independently of environmental exposure, such as active pelvic infection or pelvic malignancy.

### 2.2 Controlled ovarian stimulation and oocyte retrieval

To ensure reproducibility, factors such as stimulation protocols, trigger strategies, aspiration parameters, and sample-contact materials should be transparently documented, as tubing composition, collection interfaces, and fluid pathways may influence contamination profiles. In this pilot study, emphasis was placed on minimizing post-collection contamination and maintaining a consistent workflow throughout sampling and laboratory processing.

### 2.3 Collection, handling, and storage

Immediately following aspiration, FF samples designated for analysis were processed using a plastic-reduced protocol. Glass and metal interfaces were preferred where feasible, polymer-based disposables were minimized, and open-air exposure was limited. Samples were aliquoted into pre-rinsed glass containers, sealed, and stored at −20 °C until further processing. All handling was performed in a designated clean area separate from routine laboratory activity to reduce the risk of airborne contamination.

### 2.4 Digestion for organic matrix removal

After thawing, 10 ml from each sample was processed under a laminar-flow hood. Organic material was digested using 10% potassium hydroxide (KOH) at an approximate sample-to-reagent ratio of 1:4 and incubated overnight (approximately 16 hours) at 60 °C. KOH digestion is widely applied in biological matrices, allowing effective protein and lipid degradation while preserving most common polymers under moderate conditions. However, potential polymer-specific susceptibility (e.g., certain polyamides or rayon fibers at higher temperatures) should be considered when interpreting results.

### 2.5 Filtration and filter handling

Individually digested samples were vacuum-filtered through 1.6 µm glass-fiber filters (pre-baked or pre-rinsed according to laboratory standard operating procedures). Filters were subsequently rinsed with pre-filtered deionized water to remove residual salts, covered, and air-dried in a dust-controlled environment. All handling tools, including filter cassettes and forceps, were made of glass or metal. Although filter blanks (unused filters exposed to laboratory conditions) can be used to assess airborne contamination, this study prioritized procedural blanks.

### 2.6 Contamination control and blanks

Strict contamination-control measures were implemented throughout all stages of sample handling and analysis. Procedural blanks, consisting of filtered and processed reagent controls, were included in each batch to monitor potential contamination. Filters were initially screened using optical microscopy to identify candidate particles based on morphology (fragment, fiber, or film), size, and contrast. Selected particles were analyzed using confocal micro-Raman spectroscopy equipped with a 532 nm excitation laser. Spectra were collected over the range of 200–3400 cm^-1^ using a maximum laser power of 10 mW, an acquisition time of 3 s, and 15 accumulations per spectrum. Baseline correction was performed before spectral matching. Polymer identification was performed by comparison with the KnowItAll^®^ spectral library (Wiley, USA) using a predefined Hit Quality Index (HQI) threshold of ≥80%, together with confirmation of at least three characteristic Raman bands. The selected HQI threshold is consistent with current recommendations for Raman-based MP identification and acknowledges that environmentally weathered polymers may exhibit lower spectral similarity because of ageing, additives, and surface degradation (12, 16). These analytical criteria and reporting practices are consistent with current recommendations for Raman-based MP identification. Representative Raman spectra together with diagnostic peak assignments for the identified polymers are provided in the **Supplementary Material**. Only particles fulfilling all predefined QA/QC criteria, including acceptable spectral quality, HQI ≥80%, and confirmation of at least three diagnostic Raman bands, were classified as MPs. Spectra dominated by fluorescence or lacking characteristic polymer peaks were excluded from further analysis.

### 2.7 Particle screening and Raman spectroscopic identification

Candidate particles identified during optical screening were systematically subjected to Raman spectroscopic analysis as described above. Only particles meeting both spectral matching criteria and diagnostic peak confirmation were classified as MPs. During microscopic screening, particle morphology (fragment or fiber), color, and approximate particle dimensions were recorded before Raman analysis. Because the study was designed as a qualitative presence-detection pilot, quantitative burden estimates (particles/mL) were not performed. To minimize contamination, glass and metal laboratory equipment were used whenever possible, polymer-based consumables were minimized, samples were processed under laminar-flow conditions, and procedural blanks accompanied every analytical batch.

### 2.8 Outcomes and analysis

The primary outcome was the proportion of FF samples containing at least one Raman-confirmed MP particle. The secondary outcome was the qualitative polymer profile, defined as the range of polymer types detected across positive samples. Polymer identification required both satisfactory library matching and manual verification of characteristic Raman peaks to reduce false-positive assignments. Given the exploratory nature and limited sample size of this pilot study, analyses were descriptive. Quantitative burden assessment (e.g., particles per milliliter), particle size distribution analysis, and clinical correlation analyses were not performed.

## 3 Results

### 3.1 Patient characteristics

The study population had a median age of 39 years (interquartile range [IQR]: 34–40.5) and a median body mass index (BMI) of 26.3 kg/m² (IQR: 22.6–30.6), indicating a predominantly middle-aged cohort with a tendency toward overweight. Nine patients were diagnosed with hypothyreosis and three with insulin resistance. Reproductive history included a gravidity of 13 and a parity of 7. Detailed patient characteristics are presented in **Table 1**.

**Table 1:** Patient characteristics.

Characteristic	Value
Age (years)	39 [34-40.5]
BMI (kg/m²)	26.3 [22.6-30.6]
Hypothyreosis, n	9
Insulin resistance, n	3
Gravidity, n	13
Parity, n	7

Data are presented as median [interquartile range] or number of patients (n). BMI, body mass index.

### 3.2 Particle characteristics

MPs were detected in 22 of 27 FF samples (81.5%), while five samples showed no detectable particles. In positive samples, between one and three MP particles were identified per sample based on combined optical microscopy and Raman spectroscopic confirmation. The detected particles represented multiple polymer types, suggesting exposure to diverse sources. Among the study cohort, six of 27 patients demonstrated a viable pregnancy at 8 weeks of gestation (**Table 2**).

**Table 2:** Microplastics in follicular fluid.

Metric	Value
Total samples analyzed	27
Samples with ≥ 1 MP	22 (81.5%)
MPs per positive sample	1–3
Polymers identified	PP, PE, PA, PS, PET
Procedural blanks	no MPs detected
Pregnancy rate	6/27 (22.22%)
Raman -confirmed particles	60
Particle morphology	53 fragments, 7 fibers
Predominant particle size	<100 μm (74%)

Data are presented as number (percentage) or range, as appropriate. MPs, microplastics; PP, polypropylene; PE, polyethylene; PA, polyamide; PS, polystyrene; PET, polyethylene terephthalate.

### 3.3 Particle morphology and size distribution

A total of 60 Raman-confirmed MP particles were identified across the 22 positive FF samples. Morphological analysis showed that 53 particles (88.3%) were irregular fragments, whereas 7 particles (11.7%) were fibers. Particle colors ranged from transparent to yellow, pink, red, and blue.

**Figure 1:**
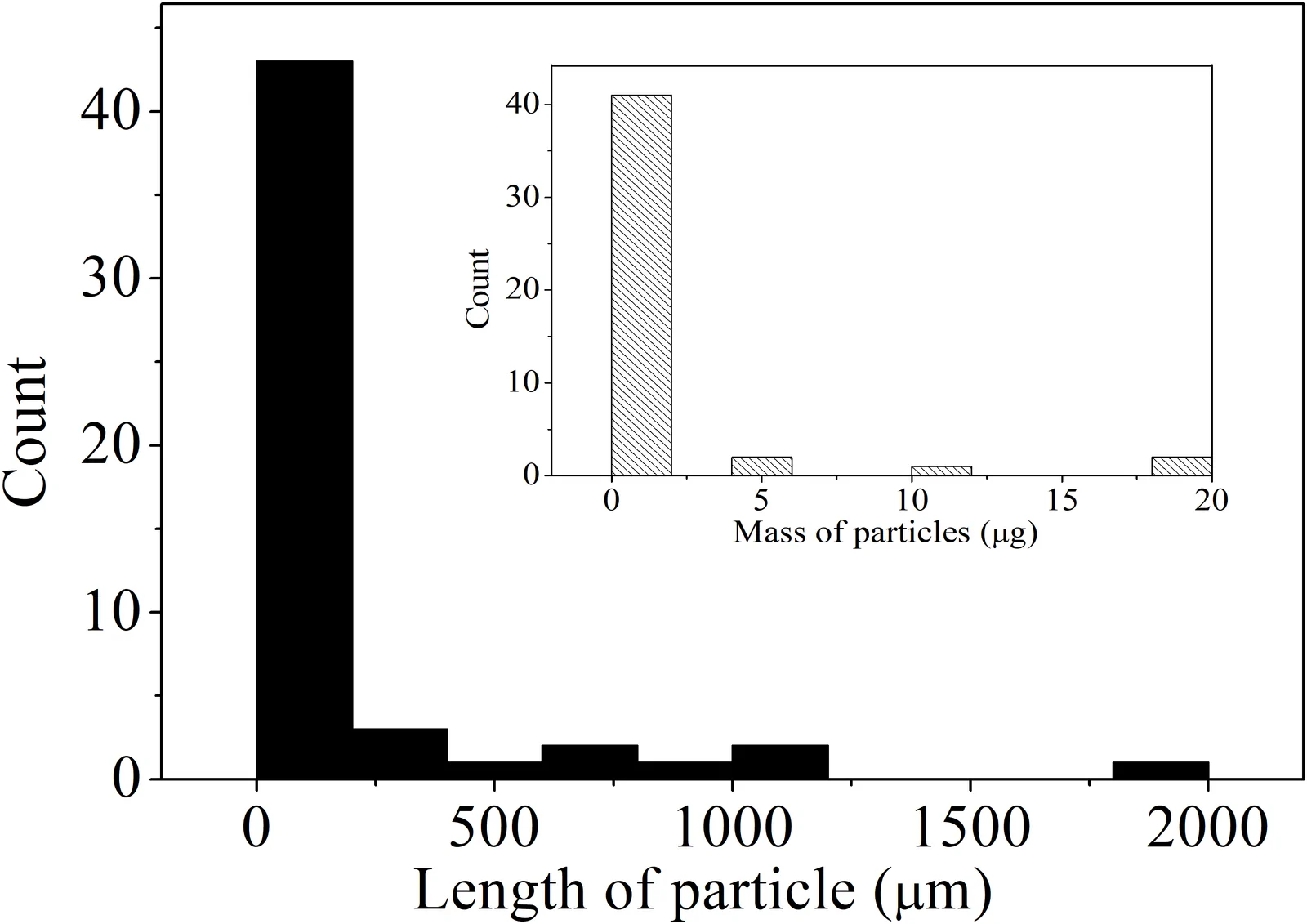
Size distribution of Raman-confirmed microplastic particles identified in human follicular fluid. Histogram showing the length distribution of the 60 Raman-confirmed microplastic particles detected in follicular fluid samples. The majority of particles (approximately 74%) measured less than 100 μm. The inset illustrates the estimated particle mass distribution based on polymer density calculations.

Particle size varied considerably, ranging from approximately 3 μm to 2 mm. Most particles (approximately 74%) measured less than 100 μm in their longest dimension (**Figure 1**). The estimated particle mass was generally low, with the majority of particles corresponding to only a few micrograms based on polymer density calculations. The mass of the particles was typically a few micrograms (calculated based on an average density of 1.4 g/cm³; see inset graph).

**Figure 2:**
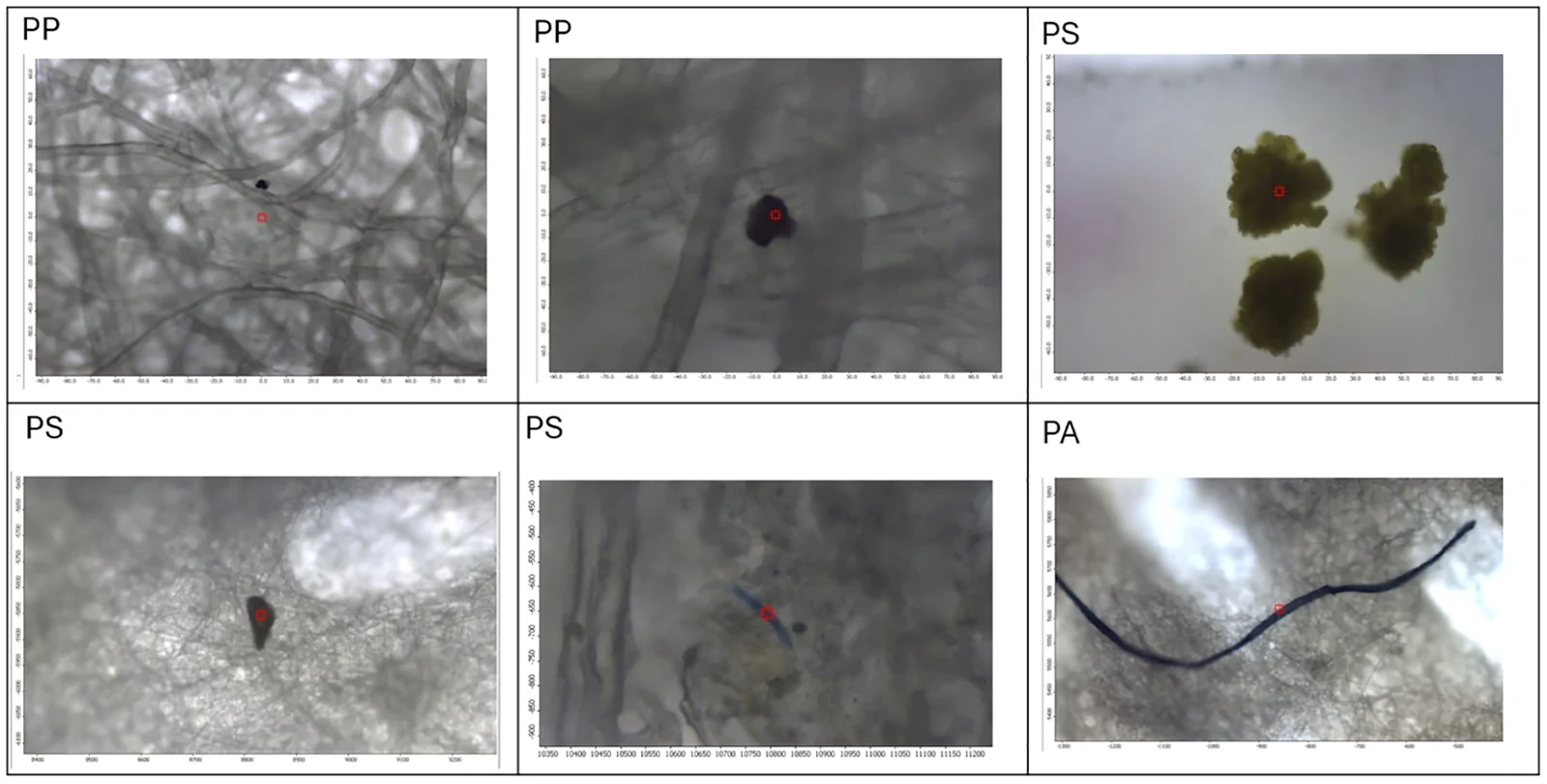
Representative optical microscopy images of microplastics identified in human follicular fluid. Representative particles detected in follicular fluid samples following digestion and filtration are shown. Polymer types were identified by confocal micro-Raman spectroscopy and are indicated in each panel (PP, polypropylene; PS, polystyrene; PA, polyamide). Red markers indicate the locations selected for Raman spectral acquisition. Particles exhibit heterogeneous morphology, including fragments and irregular aggregates, embedded within the filter matrix background.

Representative optical microscopy images and corresponding Raman-confirmed polymer identification are presented in **Figure 2**, while the overall particle size distribution is shown in **Figure 1**.

### 3.4 Polymer composition

The identified polymers included PP, which was the most frequently detected, as well as PE, PA, PS, and PET (**Figure 2**). These polymers are commonly used in environmental and consumer products.

Among the six patients who achieved a viable pregnancy, PET particles were detected in four samples. In one sample, no MPs were identified, while in another, a combination of PE and PP particles was observed. Given the limited sample size, no conclusions can be drawn regarding potential associations between MP presence and reproductive outcomes.

## 4 Discussion

### 4.1 Principal findings

The novelty of the present study lies not in demonstrating the existence of MPs in FF, but in providing an independently validated, contamination-controlled Raman spectroscopy workflow together with transparent methodological reporting that may contribute to the harmonization of future reproductive MP research. In this contamination-controlled pilot study, MPs were detected in the majority (81.5%) of human FF samples using Raman spectroscopy. The identified polymer spectrum—polypropylene (PP), polyethylene (PE), polyamide (PA), PS, and polyethylene terephthalate (PET)—reflects commonly used high-volume plastics encountered in environmental and consumer contexts (1–4, 13).

Although the number of particles per sample was low (1–3), their consistent detection in the presence of negative procedural blanks supports the analytical reliability of the workflow and demonstrates the feasibility of MP identification in FF collected during routine IVF procedures. In addition to confirming the presence of MPs, the present study provides further characterization of the recovered particles by describing their morphology, size distribution, and polymer composition. Most particles were irregular fragments smaller than 100 μm, although fibers were also observed. While these observations remain descriptive, they provide additional information regarding the characteristics of MPs present within the follicular microenvironment and may facilitate comparison with future studies adopting standardized analytical workflows.

### 4.2 Context within the literature

The present findings should be interpreted within a rapidly evolving but still methodologically heterogeneous field. MPs have been detected in multiple human biological matrices, including placenta, blood, breast milk, semen, and testicular tissue, supporting systemic exposure and tissue distribution (1–4).

Recent work has increasingly focused on the female reproductive system. Early experimental evidence demonstrated the presence of MPs in both human and bovine FF and suggested adverse effects on oocyte competence (8). Subsequent human studies confirmed these findings. Montano et al. identified MPs in FF from IVF patients, with exploratory associations involving follicle-stimulating hormone (FSH) (9), while Ni et al. reported a broader spectrum of particles using complementary analytical techniques, emphasizing methodological variability (10).

More recent studies have strengthened the clinical relevance of these observations. Si et al. reported associations between FF micro- and nanoplastics and diminished ovarian reserve, with links to granulosa-cell dysfunction and signaling pathways (11). In addition, emerging evidence indicates that MPs may accumulate in reproductive tissues and fluids more broadly, supporting the concept of systemic distribution and tissue-specific deposition (13, 14).

Taken together, these studies consistently demonstrate that MPs are detectable in human FF. However, differences in analytical platforms, contamination-control procedures, particle identification criteria, and reporting standards continue to limit direct comparison between studies. Consequently, the current challenge in this field is no longer demonstrating the presence of MPs, but improving methodological harmonization and reproducibility to enable reliable comparison across laboratories and future multicenter investigations (12).

### 4.3 Potential exposure sources and pathways

The detection of multiple polymer types suggests multifactorial exposure pathways. Potential sources include dietary intake (e.g., packaging-derived PP, PE, PET), inhalation of airborne particles, and possible iatrogenic contributions from medical materials (1, 12, 13).

Recent exposure studies indicate that ingestion and inhalation represent the dominant routes of human MP uptake, with subsequent translocation into systemic circulation and peripheral tissues (13, 15). Although a plastic-reduced protocol was applied during sample handling, pre-analytical exposure—both environmental and clinical—cannot be excluded.

Future studies should incorporate standardized exposure assessment strategies, including lifestyle questionnaires and environmental profiling, to better characterize individual exposure patterns.

### 4.4 Biological interpretation of the present findings

The detection of MPs in FF should primarily be interpreted as evidence that plastic particles can reach the ovarian follicular compartment rather than as evidence of reproductive toxicity. FF represents the immediate extracellular environment of the developing oocyte and reflects exchanges between the systemic circulation and the metabolically and hormonally active follicular cells. Consequently, the identification of MPs within this compartment establishes biological proximity to structures involved in follicular development and oocyte maturation.

Nevertheless, the present study was not designed to determine whether the detected particles interact with granulosa cells, alter the biochemical or endocrine composition of FF, or affect oocyte competence. Experimental studies provide biological plausibility for such effects through oxidative stress, mitochondrial dysfunction, altered intracellular signaling, and granulosa-cell injury (6–8, 14, 16), but these observations cannot be directly extrapolated to the low particle numbers identified in human FF in the present study. Our findings should therefore be regarded as an analytical and exposure observation that provides a rationale for future studies integrating quantitative MP measurements with endocrine, cellular, and reproductive endpoints.

### 4.5 Methodological strengths and limitations

This study has several methodological strengths, including a contamination-controlled workflow, the use of procedural blanks, and polymer-specific identification via Raman spectroscopy. Although the presence of MPs in FF has now been reported by several independent groups, considerable variability remains in sampling procedures, analytical workflows, and QA/QC strategies. We therefore believe that transparent reporting of contamination-control measures and Raman identification criteria represents an important methodological contribution that may facilitate future standardization and interlaboratory reproducibility. However, important limitations must be acknowledged. The small sample size and qualitative design preclude causal or clinical interpretation. No quantitative burden assessment, particle size distribution, or correlation with reproductive outcomes was performed.

In addition, Raman spectroscopy may underestimate total MP burden due to fluorescence interference and reduced sensitivity for smaller particles (<1–2 µm) (12). Emerging analytical frameworks highlight the importance of combining spectroscopic and mass-based techniques to improve detection accuracy (12, 15).

Future studies should therefore integrate complementary methods, including µFTIR imaging and pyrolysis–GC/MS.

### 4.6 Harmonization, QA/QC, and reporting priorities

The lack of methodological standardization remains a critical limitation in the field. Harmonized QA/QC protocols are essential to ensure reproducibility and comparability across studies (12, 15).

This includes detailed reporting of sampling procedures, materials, digestion protocols, filtration parameters, and spectral identification criteria. The consistent use of procedural and field blanks is particularly important given the high risk of contamination in MP analysis (12).

Current discrepancies between studies likely reflect both biological variability and methodological differences in detection platforms, particle-size thresholds, and analytical criteria. Without harmonization, the interpretation of results across studies will remain challenging.

### 4.7 Clinical and research implications

The principal contribution of the present study is methodological rather than clinical. Our findings independently support the detectability of MPs in human FF using a contamination-controlled Raman spectroscopic workflow. However, the qualitative design does not permit estimation of MP concentration or assessment of dose–response relationships, and the small cohort precludes meaningful evaluation of associations with IVF outcomes.

Future investigations should therefore move beyond presence detection toward standardized quantitative or semiquantitative assessment, including particle concentration, size distribution, morphology, and polymer composition. Multicenter studies using harmonized sampling and analytical protocols would be particularly valuable for assessing interlaboratory reproducibility and potential geographical differences in exposure profiles. Integration of these measurements with ovarian reserve parameters, follicular endocrine markers, oocyte maturity, fertilization, embryo development, implantation, and live birth could ultimately determine whether FF microplastics represent only markers of environmental exposure or have measurable reproductive significance. Recent reports of MPs in human reproductive fluids and broader reviews of their potential reproductive effects further support the need for such clinically integrated investigations (17, 18).

In parallel, comprehensive reviews have highlighted the growing body of evidence linking MP exposure to potential reproductive and endocrine effects, reinforcing the need for mechanistically oriented and clinically integrated research in this field (19).

Together, these findings underscore the importance of expanding current pilot observations into larger, standardized investigations that can clarify the biological and clinical significance of MPs within the human reproductive system.

## 5 Conclusions

Under contamination-controlled conditions, micro-Raman spectroscopy identified MPs in the majority of FF samples obtained during IVF. These findings support the growing evidence that environmental plastic particles are present within the human ovarian microenvironment.

While the clinical and endocrine implications remain uncertain, emerging data suggest potential associations with reproductive function, highlighting the importance of further investigation. Larger, standardized, multicenter studies integrating quantitative exposure assessment and mechanistic endpoints are required to clarify the role of MP in female reproductive health.

## Data Availability

The raw data supporting the conclusions of this article will be made available by the authors, without undue reservation.
