# Supplementary material for: Microplastics in human ovarian follicular fluid: a pilot Raman spectroscopy study
Source: Front Endocrinol (Lausanne). 2026 Sep 8;17:1856825. doi: 10.3389/fendo.2026.1856825 (PMC13599117; doi:10.3389/fendo.2026.1856825)
Supplement: Supplementary file 1 [file DataSheet1.pdf]

Wiley

Date/Time: 2026. 07. 10. 10:50

License Serial Number 1115435 Operator:

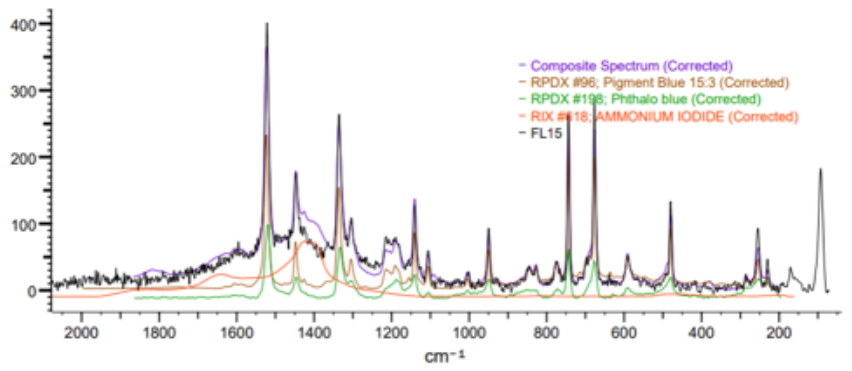

Manual Corrections: None
Ranges: Full
Search Algorithm: Correlation
Query Path: C:\Szilvi\Microplastic project\Nogyogy\15\T15_sub.SPA

Score	Info	Ratio	Name	Chemical Structure	Spectrum
90.23		N.A.	Composite Spectrum	NH_4I Tm_2O_3	
		0.51	Pigment Blue 15:3		
		0.24	Phthalo blue		
		0.17	AMMONIUM IODIDE	NH_4I	

Wiley

Date/Time: 2026. 07. 10. 10:58

License Serial Number 1115435 Operator:

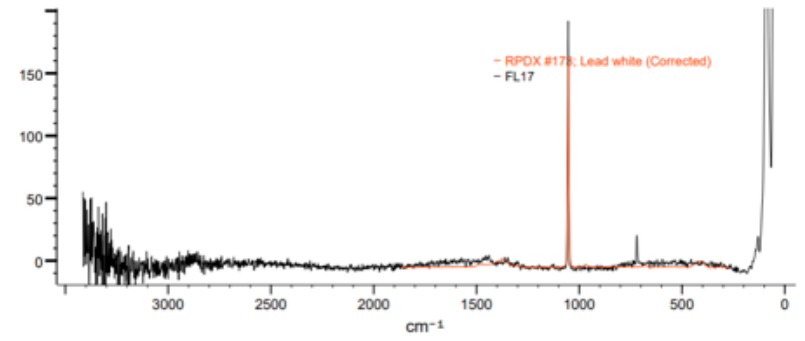

Manual Corrections: None
Ranges: Full
Search Algorithm: Correlation
Query Path: C:\Szilvi\Microplastic project\Nogyogy\17\17 001.SPA

FL17

Name	Value
Resulting HQI	77.29
Database Abbreviation	RPDX
Database Title	Raman - Pigments & Dyes - Wiley
Record ID	173
Name	Lead white
Catalog Number	46000
Chemical Description	Basic lead carbonate
Classification	Historical pigments
Color Properties	Colour Index: PW 1
Comments	It was in use since antiquity and it was the only white used in European easel paintings until the 19th century.
Instrument Name	HORIBA Xplora spectrometer
Raman Laser Wavelength	785 nm
Resolution	2 cm-1
Sample Description	Pure pigment powder
Source of Sample	Kremer
Source of Spectrum	Dr. Antonino Cosentino, Cultural Heritage Science Open Source
Synonyms	Flake white; Cremnitz white

Wiley

Date/Time: 2026. 07. 10. 11:01

License Serial Number 1115435 Operator:

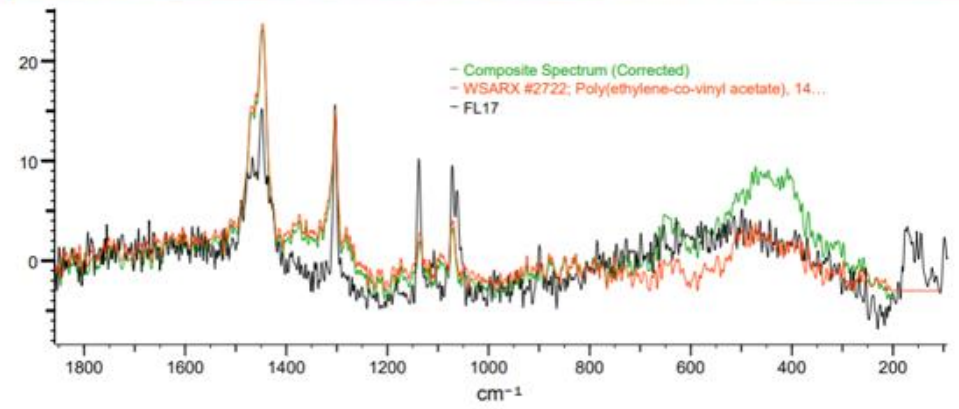

Manual Corrections: None
Ranges: Full
Search Algorithm: Correlation
Query Path: C:\Szilvi\Microplastic project\Nogyogy\17\17_002.SPA

FL17

Score	Info	Ratio	Name	Chemical Structure	Spectrum
80.03		N.A.	Composite Spectrum		
		0.81	Poly(ethylene-co-vinyl acetate), 14 wt.% vinyl acetate		

Wiley

Date/Time: 2026. 07. 10. 11:05

License Serial Number 1115435 Operator:

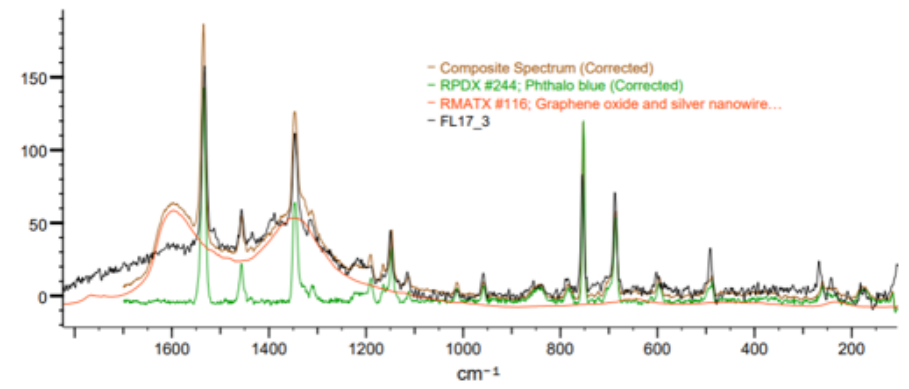

Manual Corrections: None
Ranges: Full
Search Algorithm: Correlation
Query Path: C:\Szilvi\Microplastic project\Nogyogy\17\17_005_crystal.SPA

Score	Info	Ratio	Name	Chemical Structure	Spectrum
85.12		N.A.	Composite Spectrum		
		0.70	Phthalo blue		
		0.30	Graphene oxide and silver nanowire composite		

Wiley

Date/Time: 2026. 07. 10. 11:13

License Serial Number 1115435 Operator:

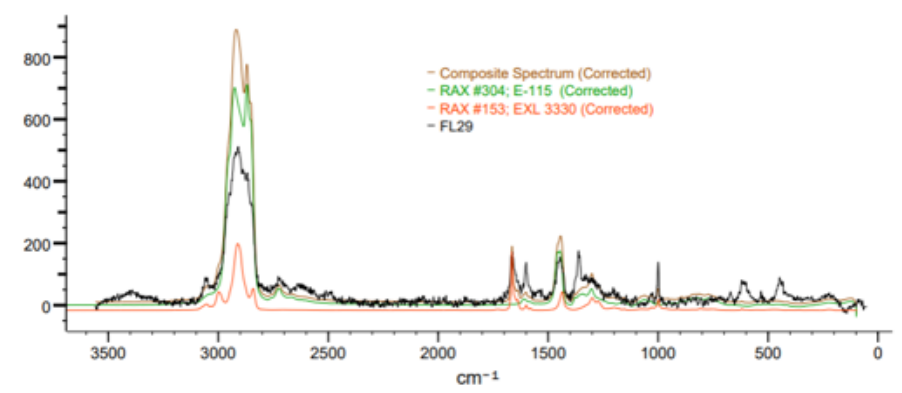

Manual Corrections: None
Ranges: Full
Search Algorithm: Correlation
Query Path: C:\Szilvi\Microplastic project\Nogyogy\29\sarga szemcse3.SPA

Score	Info	Ratio	Name	Chemical Structure	Spectrum
84.63		N.A.	Composite Spectrum		
		0.69	E-115		
		0.31	EXL 3330		

Wiley

Date/Time: 2026. 07. 10. 11:27

License Serial Number 1115435 Operator:

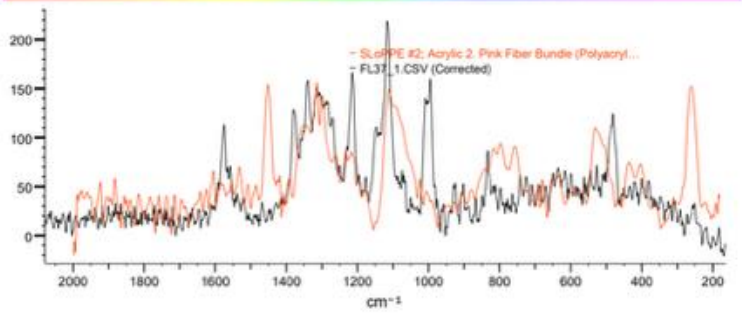

Manual Corrections: None
Ranges: Full
Search Algorithm: Correlation
Query Path: C:\Szilvi\Microplastic project\Nogyogy\37\FL37_1.CSV

Name	Value
Resulting HQI	68.39
Database Abbreviation	SLoPPE
Database Title	Spectral Library of Plastic Particles - Environmental
Record ID	2
Name	Acrylic 2. Pink Fiber Bundle (Polyacrylonitrile)
Accumulation	4
Acquisition Time	100 s
Date	07.02.2019 15:18
Delay time (s)	0 s
Detector Name	Syncerity OE
Filter	100%
Grating	600 (750nm)
Hole (µm)	300
Instrument Name	XploRA Plus
Laser (nm)	785nm_Edge
Objective	x100_VIS_LWD
Resolution	1.325 cm-1/pixel
Sample Name	Acrylic 2
Slit (µm)	100

Wiley

Date/Time: 2026. 07. 10. 11:29

License Serial Number 1115435 Operator:

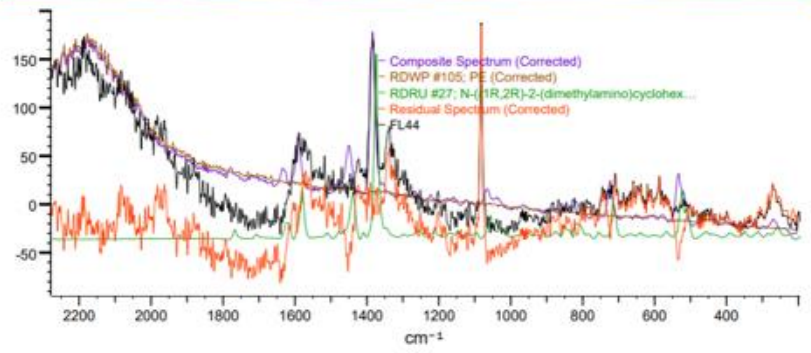

Manual Corrections: None
Ranges: Full
Search Algorithm: Correlation
Query Path: C:\Szilvi\Microplastic project\Nogyogy\44\FL44.SPA

Score	Info	Ratio	Name	Chemical Structure	Spectrum
80.39		N.A.	Composite Spectrum		
		0.52	PE		
		0.48	N-((1R,2R)-2-(dimethylamino)cyclohexyl)-N-methyl-1-naphthylcarboxamide		
		N.A.	Residual Spectrum		

Wiley

Date/Time: 2026. 07. 10. 11:42

License Serial Number 1115435 Operator:

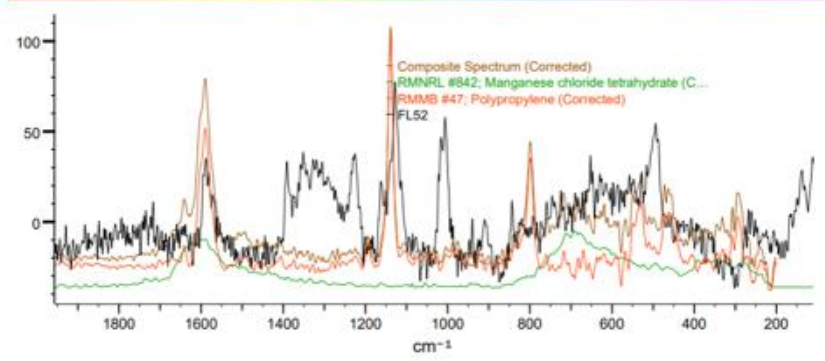

Manual Corrections: None
Ranges: Full
Search Algorithm: Correlation
Query Path: C:\Szilvi\Microplastic project\Nogyogy\52\52_002.SPA

Score	Info	Ratio	Name	Chemical Structure	Spectrum
83.04		N.A.	Composite Spectrum		
		0.83	Manganese chloride tetrahydrate		
		0.17	Polypropylene		

Wiley

Date/Time: 2026. 07. 10. 11:43

License Serial Number 1115435 Operator:

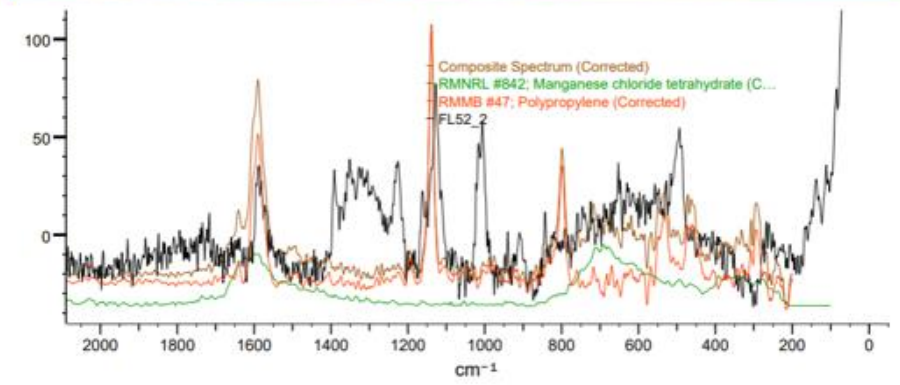

Manual Corrections: None
Ranges: Full
Search Algorithm: Correlation
Query Path: C:\Szilvi\Microplastic project\Nogyogy\52\52_002.SPA

Score	Info	Ratio	Name	Chemical Structure	Spectrum
83.04		N.A.	Composite Spectrum		
		0.83	Manganese chloride tetrahydrate		
		0.17	Polypropylene		

Wiley

Date/Time: 2026. 07. 10. 11:24

License Serial Number 1115435 Operator:

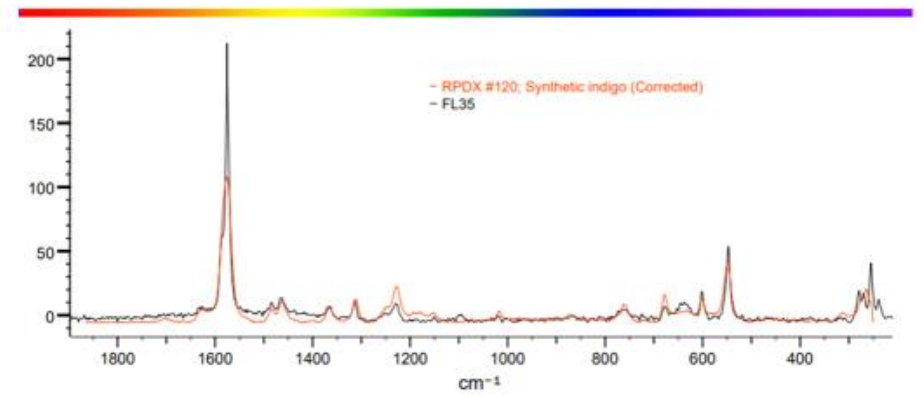

Manual Corrections: None
Ranges: Full
Search Algorithm: Correlation
Query Path: C:\Szilvi\Microplastic project\Nogyogy\35\835 1.SPA

Name	Value
Resulting HQI	77.44
Database Abbreviation	RPDX
Database Title	Raman - Pigments & Dyes - Wiley
Record ID	120
Name	Synthetic indigo
Catalog Number	36009
Color Properties	Color Index Generic Name Code: PB 66
Comments	First indigo synthesis dates back to 1882 but it was commercially produced from 1901.
Raman Laser Wavelength	830 nm
Source of Sample	Kremer
Source of Spectrum	Dr. Antonino Cosentino, Cultural Heritage Science Open Source

Wiley

Date/Time: 2026. 07. 10. 11:36

License Serial Number 1115435 Operator:

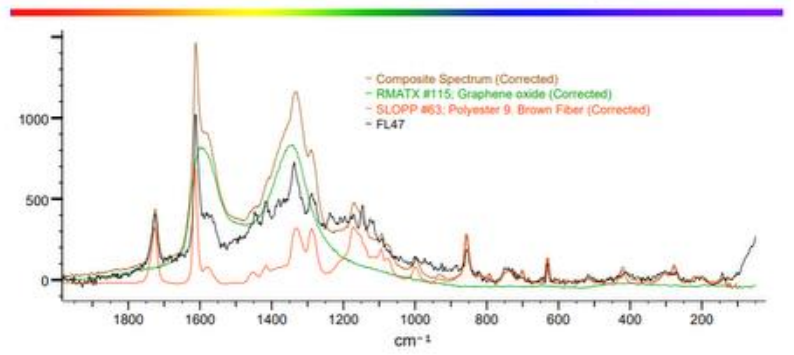

Manual Corrections: None
Ranges: Full
Search Algorithm: Correlation
Query Path: C:\Szilvi\Microplastic project\Nogyogy\47\hosszú szál_50X_polyester!.SPA

Score	Info	Ratio	Name	Chemical Structure	Spectrum
87.58		N.A.	Composite Spectrum		
		0.55	Graphene oxide		
		0.45	Polyester 9. Brown Fiber		

Wiley

Date/Time: 2026. 07. 10. 11:31

License Serial Number 1115435 Operator:

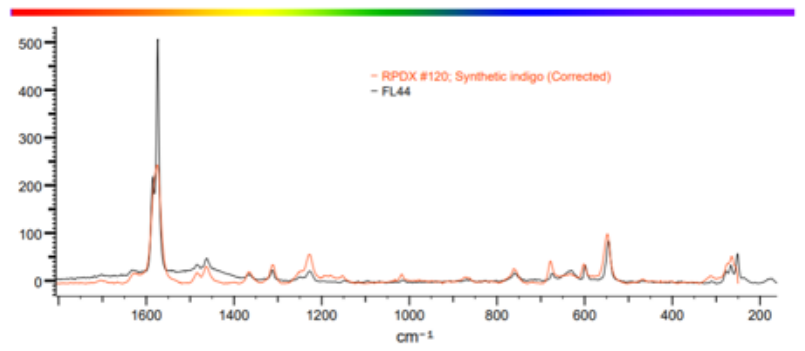

Manual Corrections: None
Ranges: Full
Search Algorithm: Correlation
Query Path: C:\Szilvi\Microplastic project\Nogyogy\44\FL44 2.SPA

Name	Value
Resulting HQI	73.42
Database Abbreviation	RPDX
Database Title	Raman - Pigments & Dyes - Wiley
Record ID	120
Name	Synthetic indigo
Catalog Number	36009
Color Properties	Color Index Generic Name Code: PB 66
Comments	First indigo synthesis dates back to 1882 but it was commercially produced from 1901.
Raman Laser Wavelength	830 nm
Source of Sample	Kremer
Source of Spectrum	Dr. Antonino Cosentino, Cultural Heritage Science Open Source

Wiley

Date/Time: 2026. 07. 10. 13:01

License Serial Number 1115435 Operator:

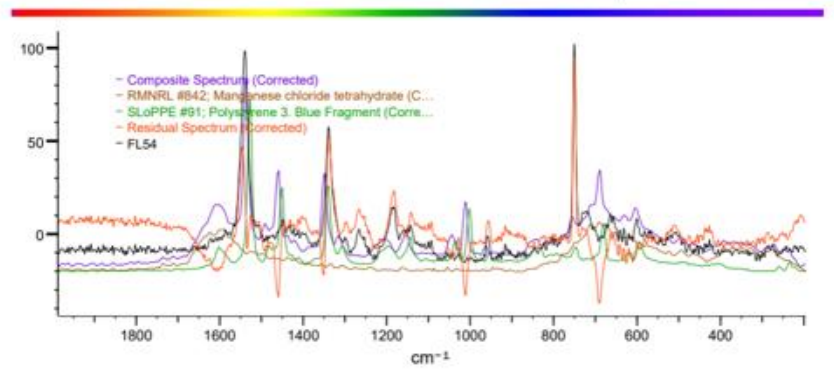

Manual Corrections: None
Ranges: Full
Search Algorithm: Correlation
Query Path: C:\Szilvi\Microplastic project\Nogyogy\54\T54_4.SPA

Score	Info	Ratio	Name	Chemical Structure	Spectrum
78.06		N.A.	Composite Spectrum		
		0.86	Manganese chloride tetrahydrate		
		0.14	Polystyrene 3. Blue Fragment		
		N.A.	Residual Spectrum		

Wiley

Date/Time: 2026. 07. 10. 13:04

License Serial Number 1115435 Operator:

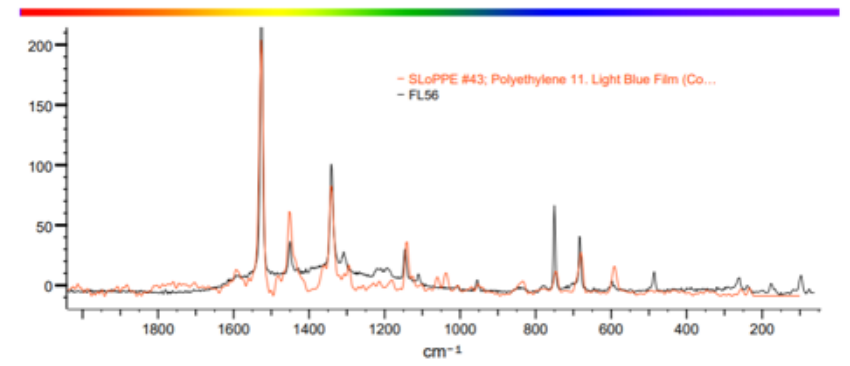

Manual Corrections: None
Ranges: Full
Search Algorithm: Correlation
Query Path: C:\Szilvi\Microplastic project\Nogyogy\56\56 003.SPA

Name	Value
Resulting HQI	71.34
Database Abbreviation	SLoPPE
Database Title	Spectral Library of Plastic Particles - Environmental
Record ID	43
Name	Polyethylene 11. Light Blue Film
Accumulation	4
Acquisition Time	100 s
Date	02.01.2019 13:44
Delay time (s)	0 s
Detector Name	Syncerity OE
Filter	25%
Grating	1200 (750nm)
Hole (µm)	300
Instrument Name	XploRA Plus
Laser (nm)	532nm_Edge
Objective	x100_VIS_LWD
Resolution	3.252 cm-1/pixel
Sample Name	PE 11
Slit (µm)	100

Wiley

Date/Time: 2026. 07. 10. 13:08

License Serial Number 1115435 Operator:

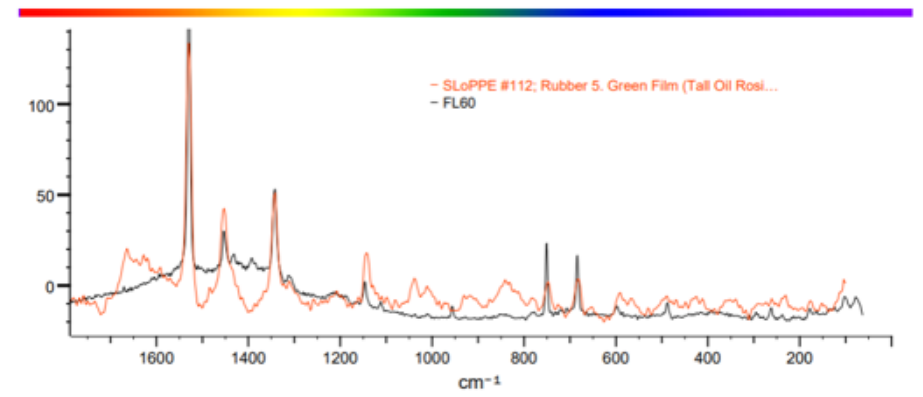

Manual Corrections: None
Ranges: Full
Search Algorithm: Correlation
Query Path: C:\Szilvi\Microplastic project\Nogyogy\60\60 009.SPA

Name	Value
Resulting HQI	64.10
Database Abbreviation	SLoPPE
Database Title	Spectral Library of Plastic Particles - Environmental
Record ID	112
Name	Rubber 5. Green Film (Tall Oil Rosin)
Accumulation	10
Acquisition Time	100 s
Date	07.03.2019 16:18
Delay time (s)	0 s
Detector Name	Syncerity OE
Filter	25%
Grating	1200 (750nm)
Hole (µm)	300
Instrument Name	XploRA Plus
Laser (nm)	532nm_Edge
Objective	x100_VIS_LWD
Resolution	3.252 cm-1/pixel
Sample Name	Rubber 5
Slit (µm)	100
